# Surface Morphology Evolution during Chemical Mechanical Polishing Based on Microscale Material Removal Modeling for Monocrystalline Silicon

**DOI:** 10.3390/ma15165641

**Published:** 2022-08-17

**Authors:** Jingjing Xia, Jun Yu, Siwen Lu, Qiushi Huang, Chun Xie, Zhanshan Wang

**Affiliations:** 1 MOE Key Laboratory of Advanced Micro-Structured Materials, Institute of Precision Optical Engineering (IPOE), School of Physics Science and Engineering, Tongji University, Shanghai 200092, China; 2 Sino-German College of Applied Sciences, Tongji University, Shanghai 200092, China

**Keywords:** chemical–mechanical polishing, monocrystalline silicon, removal mechanism, roughness, power spectral density

## Abstract

Chemical–mechanical polishing (CMP) is widely adopted as a key bridge between fine rotation grinding and ion beam figuring in super-smooth monocrystalline silicon mirror manufacturing. However, controlling mid- to short-spatial-period errors during CMP is a challenge owing to the complex chemical–mechanical material removal process during surface morphology formation. In this study, the nature of chemical and mechanical material removal during CMP is theoretically studied based on a three-system elastic–plastic model and wet chemical etching behavior. The effect of the applied load, material properties, abrasive size distribution, and chemical reaction rate on the polishing surface morphology is evaluated. A microscale material removal model is established to numerically predict the silicon surface morphology and to explain the surface roughness evolution and the source of nanoscale intrinsic polishing scratches. The simulated surface morphology is consistent with the experimental results obtained by using the same polishing parameters tested by employing profilometry and atomic force microscopy. The PSD curve for both simulated surface and experimental results by profilometry and atomic force microscopy follows linear relation with double-logarithmic coordinates. This model can be used to adjust the polishing parameters for surface quality optimization, which facilitates CMP manufacturing.

## 1. Introduction

Owing to its excellent mid- to short-spatial-period error control with a root-mean-square (RMS) roughness value in the subnanometer level [1], chemical–mechanical polishing (CMP) has been applied for monocrystalline silicon mirror fabrication in the aerospace industry and high energy beam system domains [2,3,4,5]. Microscale surface morphology is a direct source for silicon mirror evaluations of mid- to short-spatial-period errors during CMP. Optimization of the silicon surface morphology requires precise control of surface features, such as roughness and microscratches, which are determined based on material removal characteristics.

Preston’s equation is widely accepted for describing macrolevel material removal, where the material removal rate (MRR) is the product of Preston’s constant, the applied load, and the workpiece velocity. However, several aspects of CMP, such as slurry hydrodynamics and microlevel surface features, cannot be explained by using this linear equation. Thus, efforts have been made to expand Preston’s equation into theoretical, nonlinear forms. Runnels [6] proposed a hydrodynamic erosion model that considers the fluid layer shear stress by using the steady-state two-dimensional (2D) Navier–Stokes equation. This feature-scale model linked the wafer and abrasive particles together through the layer thickness and erosion law, with good agreement with the experimental erosion profile data. Sundararajan et al. [7] developed a 2D CMP model based on lubrication theory and the Reynolds equation. The simulated average MRR was consistent with the experimental results for different abrasive concentrations at the same applied load and velocity. Han et al. [8] reported the influence of the applied load and relative velocity on the surface roughness. The applied load and velocity on the workpiece were adjusted to improve the surface roughness from 14 to 5.9 nm. The relationship between surface roughness and macroscopic factors such as the applied load, relative velocity, and slurry viscosity was qualitatively interpreted from the perspective of contact pressure fluctuation and mechanical material removal by abrasive particles. These results provided a quantitative prediction of the macrolevel MRR; however, the microlevel material removal mechanism is not yet completely understood.

Elastic–plastic deformation on an assumed thick surface layer activated by a chemical reaction has typically been used to simulate the material removal process during CMP [9,10]. The geometry and dimensions of the mechanical material removal area for a single abrasive can be derived from the abrasive size and mechanical parameters, such as Young’s modulus [11]. Hence, the total MRR is a function of the abrasive size distribution (ASD) and pad geometry. However, this mechanical material removal model is limited in explaining either the MRR deviation for different slurry pH values or the material removal mechanism by pure water. Therefore, multiple attempts have been made to estimate the full chemical–mechanical MRR. Luo et al. [12,13] developed a formulation to predict the MRR by introducing the fitted wafer hardness combining the chemical–mechanical characteristics in the Hertzian contact theory [14,15]. The predicted MRR showed good consistency for different abrasive sizes and ASD standard deviations. Suratwala et al. [15,16,17,18] extended Luo’s model by applying fitted chemical material removal in a single-abrasive material removal volume. The simulated surface morphology captured salient features such as roughness, surface texture, and power spectral density (PSD). With a molecular removal depth of 0.04 nm under chemical effects, the calculated MRR was close to the experimental value. Akbar et al. [19,20] modeled the chemical effects during CMP as the diffusion of water into the workpiece. The moving reacted and unreacted sites have different hardness linked to plastic deformation and surface profile for scratching in the next polishing iteration. A linear dependence of MRR on the applied load was obtained with the predicted MRR slightly smaller than the experimental results. Lee et al. [21] further investigated the thermal effect on the chemical reactions during CMP on a copper wafer by conducting a static etch test. The total MRR model was established by combining the chemical–mechanical removal rate using the modified Preston equation and the dynamic etching rate using the Arrhenius equation. These models are helpful for understanding the material removal characteristics. However, most of the previous research focused on the wafer-scale material removal process. The chemical reaction was either approximated to have similar effects on the microscale surface texture as mechanical material removal, or was combined with mechanical parameters such as workpiece hardness. Furthermore, research on the simulation of microscale surface morphology is limited.

In this study, we propose a microscale surface morphology evolution model that incorporates the effects of the applied load, material properties, ASD, and chemical reaction by employing three-system elastic–plastic deformation and wet chemical etching. A polishing experiment was performed to verify the surface morphology from the perspective of the RMS roughness evolution and PSD function. We expect that our results could be useful for investigations on microscale material removal mechanisms during CMP and for improving the super-smooth silicon mirror surface quality.

## 2. Micropolishing Model

The surface morphology of the polishing workpiece is determined by the chemical and mechanical material removal processes during CMP. In this section, we discuss the monocrystalline silicon material removal characteristics of these two processes based on the Hertzian contact [14,15] and wet chemical etching models. A micropolishing model combining the two models is proposed to quantitatively explain the roles of the ASD, number density, applied load, and chemical etching rate, together with workpiece, abrasive, and pad characteristics.

### 2.1. Mechanical Material Removal by Plastic Deformation

**Figure 1:**
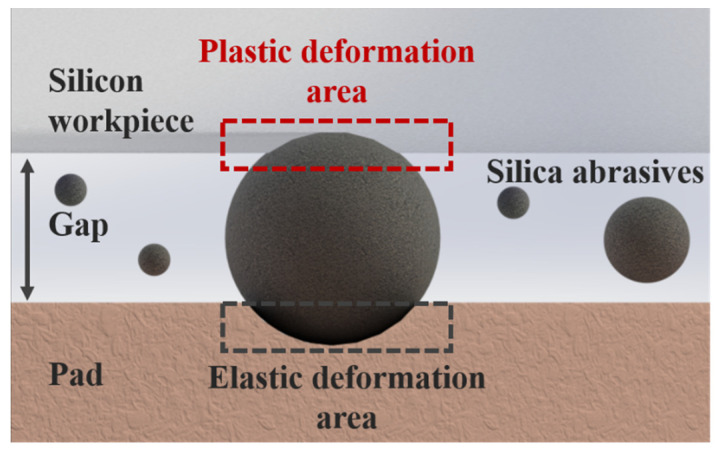
Profile of free abrasives entering the gap between the pad and silicon workpiece. Abrasives, such as silica, under a dynamic load generate recoverable elastic deformation on the pad and permanent plastic deformation on the ductile silicon workpiece. Continuous plastic deformation creates scratches on the workpiece surface.

**Figure 2:**
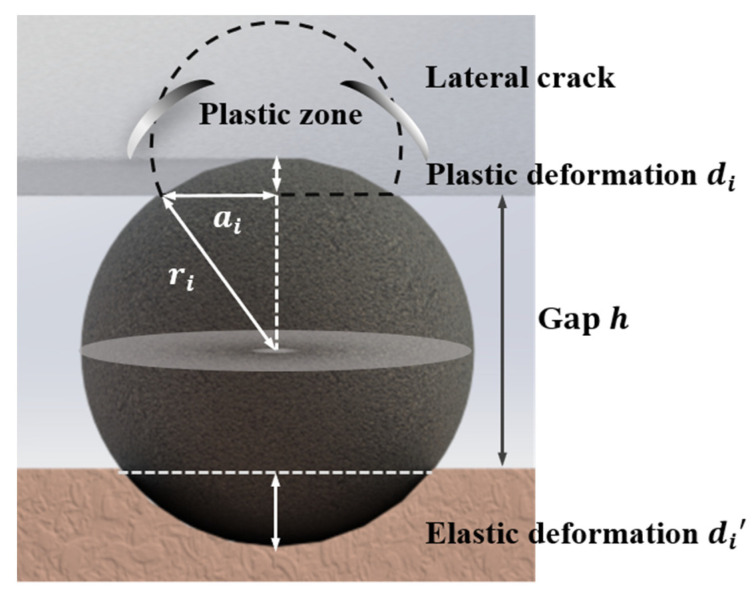
Schematic of plastic deformation and lateral cracks on the silicon workpiece by single abrasive at the pad–workpiece interface; di and di’ denote the workpiece plastic and pad elastic deformation depths, respectively, h is the gap between the pad and workpiece, $a_{i}$ is the contact area radius for the abrasive and workpiece, and $r_{i}$ is the radius of the abrasive particle.

**Figure 3:**
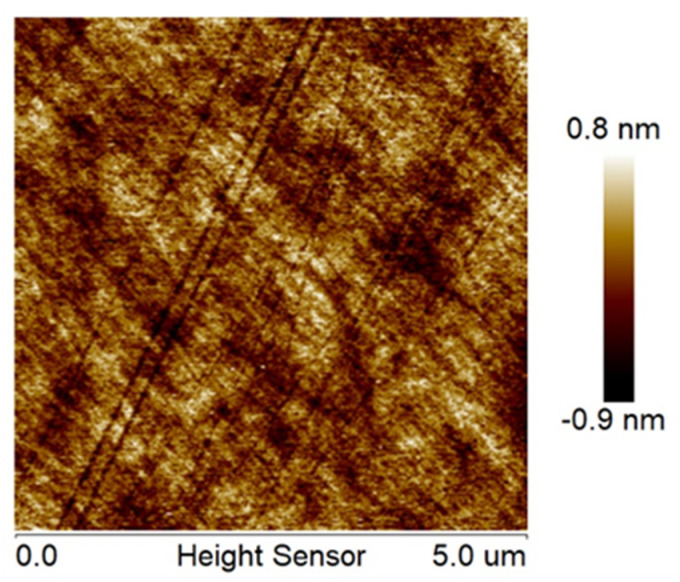
Silicon surface morphology of silicon determined by using atomic force microscopy after CMP. Isotropic scratches and textures can be clearly observed with an RMS roughness of 0.247 nm within a 5 × 5 μm^2^ area.

During CMP, free abrasives with diameters 10–100 nm in a polishing slurry enter the gap between the polishing pad and workpiece (Figure 1) under a dynamic load of 0.1–10 μN per particle [11,13]. Most adopted pad materials, such as pitch and polyurethane, exhibit elastic behavior. Contact abrasives generate recoverable elastic indentations within the elastic limit. For brittle workpieces, such as silicon and carbide silicon, both brittle and ductile abrasion exist [22,23]. Sphere-shaped abrasives create nanoscale scratches by plastic deformation with lateral cracks (Figure 2); this is referred to as the mechanical material removal process in CMP [24]. This microscratching or microcracking process by a high number of free abrasives is similar to a dynamic blunt indent on the workpiece [13,23]. The typical nanoscale scratch depth generated in this process is 0.1–1 nm for brittle materials such as silicon, which is 2–3 orders of magnitude smaller than the silica abrasive size. The final surface morphology of the silicon workpiece is partially determined by this scratching process (Figure 3).

To simulate the evolution of the surface morphology during CMP, a reliable scratch geometry is required. The actual deformation for the plastic–elastic behavior during scratching includes elastic, plastic, and densification deformation. The last two items are the permanent deformation on the silicon surface. In the mechanic material removal model, we focused on the relationship between applied load and the morphology of the scratch. To simplify the problem, scratching was assumed to be the accumulation of Hertzian cracks along the movement direction of abrasive particles [14,15,19,20]. First, we considered single abrasive particles in contact with the pad and the workpiece. To simplify the plastic–elastic behavior for a pad–abrasive–workpiece three-body system, we adopted Suratwala’s [15] hypothesis that the formation of applied load is determined by elastic contact mechanics. According to Johnson [14], the combined modulus $E_{eff}$ of the system satisfies [14,15]

(1) $$E_{eff}=\frac{E_{m-p}E_{l-p}}{{\left(E_{m-p}^{2/3}+E_{l-p}^{2/3}\right)}^{\frac{3}{2}}},$$

(2) $$\frac{1}{E_{m-p}}\equiv \frac{1-\nu_{m}^{2}}{E_{m}}+\frac{1-\nu_{p}^{2}}{E_{p}},$$

(3) $$\frac{1}{E_{l-p}}\equiv \frac{1-\nu_{l}^{2}}{E_{l}}+\frac{1-\nu_{p}^{2}}{E_{p}},$$

where $\nu_{m}$, $\nu_{p}$, and $\nu_{l}$ denote the Poisson ratios for the workpiece, polishing abrasive, and pad, respectively; $E_{m}$, $E_{p}$, and $E_{l}$ the Young’s moduli, respectively; and $E_{m-p}$ and $E_{l-p}$ are the composite moduli for the workpiece–slurry and pad–slurry interfaces, respectively. Sphere-shaped abrasives produce the classical Hertzian cone crack as the silicon workpiece manifests ductile behaviors and produces plastic flow of material [23,25]. Based on the Hertzian contact theory, the radius $a_{i}$ of the contact circle is given by [14,15]

(4) $$a_{i}={\left(\frac{3P_{i}r_{i}}{4E_{eff}}\right)}^{\frac{1}{3}},$$

where $P_{i}$ denotes the applied load for a single particle and $r_{i}$ denotes the radius of the abrasive. The abrasive and plastic deformation areas shown in Figure 2 have a geometric relationship given by

(5) $$a_{i}{}^{2}+{\left(r_{i}-d_{i}\right)}^{2}=r_{i}{}^{2},$$

where $d_{i}$ represents the penetration depth of the abrasive. The plastic deformation depth $d_{i}{}^{m-p}$ satisfies [14,15]

(6) $$d_{i}{}^{m-p}=d_{i}{\left(\frac{E_{eff}}{E_{m-p}}\right)}^{\frac{2}{3}}.$$

where $d_{i}$ is approximately three orders of magnitude smaller than $a_{i}$ during CMP; therefore, Equation (5) can be rewritten as

(7) $$a_{i}{}^{2}\approx 2r_{i}d_{i}.$$

By substituting Equation (7) into Equation (4), we can obtain the applied load as a function of the abrasive size:

(8) $$P_{i}=\frac{8\sqrt{2}E_{eff}}{3}{\left(d_{i}{}^{3}r_{i}\right)}^{\frac{1}{2}}.$$

For free abrasives, the total applied load is the sum of every abrasive participating in material removal, or more generally, the integration of the product of the applied load and number density within each abrasive size range, as follows:

(9) $$P_{total}=\int P_{i}N_{i}dr_{i}=\int P_{i}N_{t}\;\cdot \;f\left(r_{i}\right)dr_{i},$$

where $N_{i}$ is the number of abrasives of radius $r_{i}$, $f\left(r_{i}\right)$ is the probability density for the ASD curve of radius $r_{i}$, and $N_{t}$ is the total number of abrasives at the workpiece–pad interface, which is determined by the slurry concentration. For the polishing slurry, the ASD can be fitted by a logarithmic normal distribution in the following form [9,13]:

(10) $$f\left(r_{i}\right)=\frac{1}{\sqrt{2\pi }\sigma }e^{{\left(\frac{-\left(log(r_{i})-r_{0}\right)}{\sqrt{2}\sigma }\right)}^{2}},$$

where $r_{0}$ denotes the average abrasive size and σ denotes the half width of the ASD. The abrasives should directly contact the pad and workpiece to form plastic deformation, indicating that the size of the abrasives should be larger than the gap between the pad and workpiece. In addition, larger particles were not permitted to enter the gap in our experiments, indicating a maximum abrasive size restriction. Here, we assumed that the size of the effective abrasives should satisfy the following relationship:

(11) $$\frac{h}{2}<r_{i}<r_{0}+3\sigma ,$$

where *h* is the gap between the workpiece and pad, and $r_{0}$ and σ denote the mean value and standard deviation from the ASD fitting result, respectively. Abrasives with radii beyond this range cannot create microscratches and do not contribute to the applied load; hence, they can be removed from the integral. Equations (8)–(11) can be combined to rewrite $P_{total}$ as a function of h:

(12) $$P_{total}=N_{t}\int_{\frac{h}{2}}^{r_{0}+3\sigma }\frac{8\sqrt{2}E_{eff}}{3}{\left[{\left(2r_{i}-h\right)}^{3}r_{i}\right]}^{\frac{1}{2}}\times \frac{1}{\sqrt{2\pi }\sigma }e^{{\left(\frac{-\left(log\left(r_{i}\right)-r_{0}\right)}{\sqrt{2}\sigma }\right)}^{2}}dr_{i}.$$

From the simple elastic load balance by the pad and workpiece over the abrasive, we obtained the gap value, contact radius, abrasive penetration depth, and plastic deformation depth corresponding to the experiment. This formulation is based on a ductile-regime polishing hypothesis proved by Bifano [23] to be capable of brittle materials such as silicon and silicon carbide, thereby is also adaptable to ductile materials such as fused silica and soda-lime glass.

### 2.2. Chemical Material Removal by Simplified Wet Chemical Etching

Chemical material removal is another basic material removal process that occurs during CMP. In an alkaline atmosphere, which is widely applied for silicon mirror CMP, silicon is considered to be removed through the following reactions [26]:

(13) $$Si+2OH^{-}\to SiO_{2}+2H^{+}+4e^{-}$$

and

(14) $$SiO_{2}+2OH^{-}+2H^{+}\to Si{\left(OH\right)}_{4}.$$

Silicon atoms at the workpiece surface are oxidized by hydroxyl ions in the aqueous slurry and removed from the bulk material. This process is similar to the wet chemical etching of silicon in KOH [27,28], except that the reaction rate is considerably lower during CMP. Although chemical reaction is not the major part of material removal, this full-plane erosion helps expose the grain boundaries formed during formation, thereby causing orange peel structures on the silicon surface [29,30,31]. The different binding energies of the crystal planes, surfactants with micellization, and chelation make this chemical process almost impossible for precise quantification. To simplify the chemical material removal model, two assumptions were made: (1) monocrystalline silicon has a similar reaction preference as the isotropic substances. The mechanical component of the polishing rate plays a dominant role in determining the polishing rate in the boundary lubrication regime, as is our case [32]. No scratch orientation preference during CMP has yet been reported, proving that the anisotropic characteristic of silicon can be ignored for simplification of the chemical reaction during CMP; (2) the reaction rate at the scratch bottom is the same as that of bulk materials. The simulation error for this assumption can easily be eliminated by linear fitting of the expansion ratio for the RMS roughness or peak–valley value. The trends of the surface morphology evolution and salient features are not influenced.

**Figure 4:**
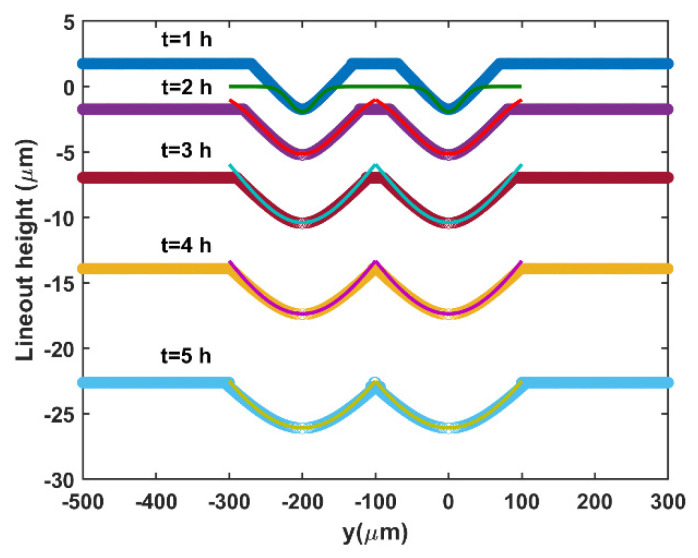
Surface area–volume model simulation of two neighboring parabolic cracks (thick line) and Gaussian fitting results (thin line) in 2D. The etching rate for bulk silica material was 1.74 μm/h [34], and the etching time was 1–5 h.

For hydrofluoric acid-based etching on fused silica, Feit [33] described the morphological evolution of two neighboring cracks by using a surface area–volume model. The width of the parabolic-shaped crack increased with the square root of the etching time before the intersection (Equation (15)). When cracks or scratches intersect with neighboring cracks, the crack widening rate decreases because the width of the coalescing cracks is twice as large. The etching widths of the two neighboring parabolic cracks are shown in Figure 4. The scratch width function for the silicon workpiece could be obtained by adjusting the etching rate $r_{e}$, the coefficient 2.8, and the initial width $w_{0}$ in Equation (15).

(15) $$w\left(t\right)=\sqrt{2.8r_{e}tw_{0}+w_{0}{}^{2}},$$

where w is the crack width, $w_{0}$ is the initial crack width, $r_{e}$ is the etching rate, t is the crack etching time, and the coefficient 2.8 is related to the initial crack shape in their model.

We simulated the evolution of 3000 randomly selected scratches on an ideal surface by using Feit’s model. The computation period for one iteration over this scratched surface was 1055 ms. Because scratches accumulated over repeated iterations, the time cost rapidly increased over the first few iterations; this is not suitable for MRR simulations during CMP.

To reduce the storage and time cost, a rapid scratch morphology generation method is needed. We proposed a Gaussian blur and smoothing method [35] for scratch widening during CMP. The MATLAB ‘fspecial’ and ‘imfilter’ command predefined a rotationally symmetric Gaussian image filter, in which the Gaussian function in 2D satisfies

(16) $$y=a\;\cdot \;exp[-{\left(\frac{x-b}{c}\right)}^{2}].\;$$

The relationship between the scratch width and etching time was established by curve fitting with different Gaussian parameters a, b, and c. The thin lines show the Gaussian fitting results in 2D, which correlate well with the thick lines obtained by using the surface area–volume model, as displayed in Figure 4. R-square for fitting is close to 1 for the etching time 1–5 h, which means a Gaussian function is qualified to describe scratches during CMP. The eroding profile can easily be obtained by fitting a and c in Equation (16) through time instead of calculating depth (d) and width (w) for each scratch. For 3000 randomly selected scratches, the computation period was 22 ms, which was 50 times less than that of the surface area–volume model. Using an image filter with a Gaussian blur kernel [35] predefined by the fitting results, we can simulate the scratch width evolution in 3D.

**Figure 5:**
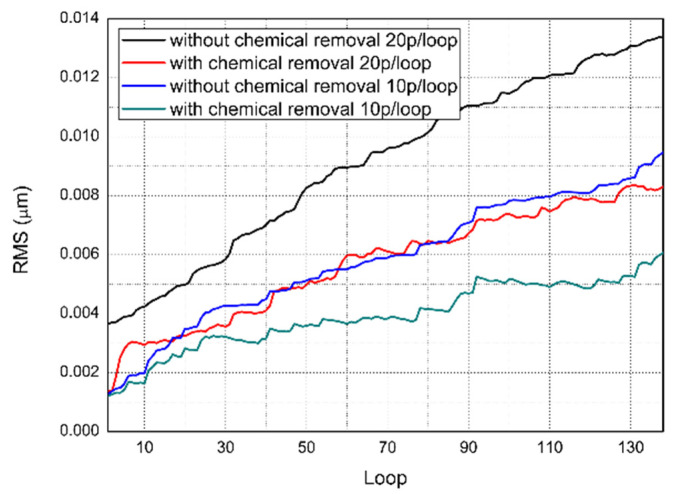
Simulated RMS roughness evolution with and without chemical material removal for 150 loops. The red and black solid lines are for 20 randomly selected particles, which are randomly placed for each loop (3000 in total), and the blue and green solid lines are for 10 randomly selected particles, which are randomly placed for each loop (1500 in total).

To define the simulated surface in numerical values, surface parameters that are best correlated to microscale surface morphology should be adopted. The simulated surface morphology consists of two parts, scratches and orange-peel texture. RMS roughness is chosen here because of its adaptability in characterizing orange peel mentioned in Rebeggiani and Miranda-Medina’s works [31,36]; it is also commonly adopted for engineering standards. Figure 5 illustrates the evolution of the surface RMS roughness for loop 150 with and without Gaussian blur by 10 or 20 randomly selected abrasive particles per loop. Despite slight vibrations due to random particle selection for each loop, the RMS roughness increased over the polishing time on an ideal surface for the four simulation conditions. The RMS roughness increased more slowly with Gaussian blur, which indicated that chemical material removal had an inhibitory effect on the surface during CMP. As the smoothing parameters were adjusted, the chemical effect gradually approached the true level. In addition, the RMS growth curve for 10 abrasives per loop without Gaussian blur had a growth rate similar to that of 20 abrasives per loop with Gaussian blur. This result implies that the abrasive number and chemical etching rate may have similar effects on the RMS roughness, even though they are associated with different material removal processes and surface features.

### 2.3. Micropolishing Model during CMP

**Figure 6:**
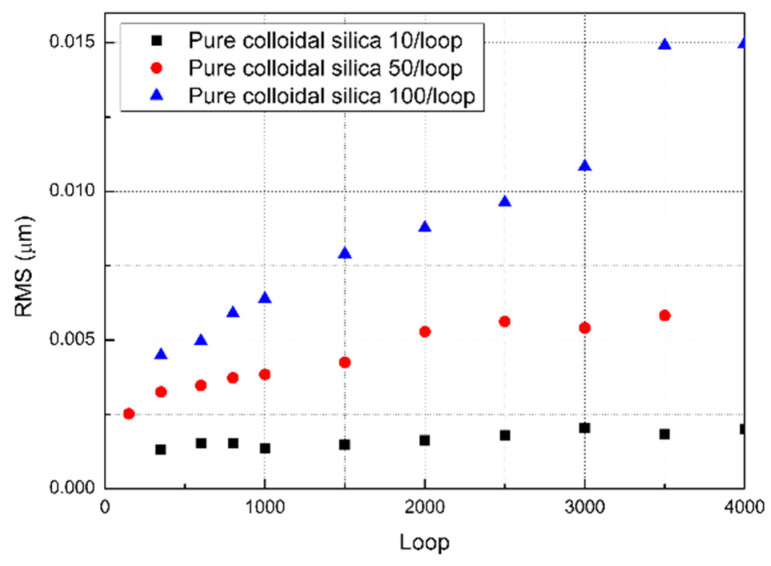
Simulated RMS roughness by pure colloidal silica with 10, 50, and 100 abrasives per loop.

The microscopic surface morphology was determined by the chemical and mechanical material removal processes discussed in the previous section. The simplest approach to combine chemical and mechanical material removal processes in a micropolishing model is to find two individual parameters in the two processes that have similar effects on the surface morphology evolution. As shown in Figure 5 in Section 2.2, both the chemical etching rate and abrasive number affect the RMS growth rate. The RMS roughness is linear to the standard deviation chosen for Gaussian blur, which is defined by the chemical etching rate. To verify the influence of the number of abrasives, we tested the RMS roughness evolution as a function of the number of abrasive particles. Chemical removal prevailed over mechanical removal at an extremely low concentration of 10 particles per loop sample (as shown in Figure 6). The RMS roughness changed slightly over the microlevel while a texture similar to an orange peel increased because of the exposure rate of grain boundaries rose, which worsened the surface quality. For a large concentration (100 particles per loop), the mechanical removal surpassed the chemical removal. Microscratches are the main features of the silicon surface. The RMS roughness inflection point appears after 3500 iterations for 100 particles per loop. For 50 particles per loop, 2500 loops were sufficient to reach the inflection point. It was concluded that for a large slurry concentration, the MRR increased with a decrease in the surface RMS roughness, which was consistent with the experimental results. Comparing Figure 5 and Figure 6, the chemical etching rate and abrasive particle number changed the RMS roughness growth rate through time in a similar manner. By normalizing the chemical etching rate and abrasive particle number, the two material removal processes can be combined to describe the surface morphology evolution.

**Figure 7:**
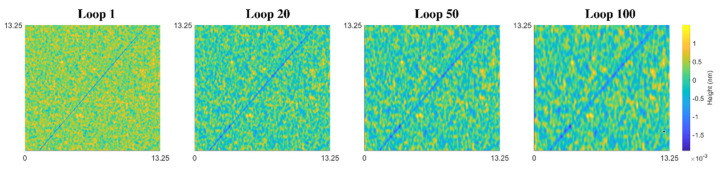
Simulated surface morphology for chemical material removal within 101 loops by randomly selected particles (sampling points: 500 × 500 within a 13.25 μm × 13.25 μm range in the x–y direction).

First, Gaussian blur over the entire surface was adopted for continuous scratching. The size of the symmetric Gaussian low-pass filter is linear to the contact circle radius $a_{i}$ of each effective abrasive for the initial scratch. The standard deviation of the filter is determined by its linear relationship with the square root of the deformation depth $d_{i}$. The scratch evolution is shown in Figure 7 for 101 loops.

**Figure 8:**
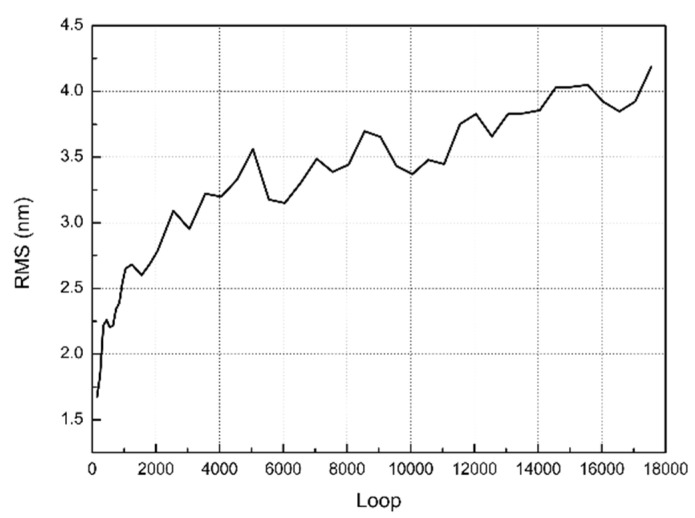
RMS roughness evolution for 17,550 loops with Gaussian blur over the entire surface. The curve is nonconvergent for one abrasive per loop with quasilinear growth after 4000 loops.

The surface morphology evolution initially approached the main problem. However, a nonconvergence RMS roughness evolution with a slight vibration owing to random particle selection, as shown in Figure 8, was obtained after extended iterations. This finding reversed the experimental result, that for specific slurry ASD, a limited RMS was reached after sufficient iterations. Gaussian blur for the entire surface led to a change in the original Gaussian-type height distribution $f\left(z\right)$, as follows:

(17) $$f\left(z\right)=\int_{-\infty }^{+\infty }f_{1}\left(x\right)f_{2}\left(z-x\right)dx=\int_{-\infty }^{+\infty }\frac{1}{2\pi \sigma_{1}\sigma_{2}}e^{-\left[\frac{{\left(x-\mu_{1}\right)}^{2}}{2\sigma_{1}^{2}}+\frac{{\left(t-x+\mu_{2}\right)}^{2}}{2\sigma_{2}^{2}}\right]}dx,\;$$

where $f_{1}$ and $f_{2}$ denote the distribution functions of the original surface height and Gaussian blur, respectively, $\sigma_{1}$ and $\sigma_{2}$ the standard deviations, and $\mu_{1}$ and $\mu_{2}$ the average values. Considering $\mu_{1}=0$ and $\mu_{2}=0$ for simplification, we obtain

(18) $$f\left(z\right)=A\;\cdot \;e^{-\frac{z^{2}}{2\sigma_{3}^{2}}},$$

where $\sigma_{3}$ denotes the standard deviation for the new surface height distribution. A and $\sigma_{3}$ satisfy

(19) $$A=\sqrt{2\pi }\sqrt{\frac{\sigma_{1}^{2}\sigma_{2}^{2}}{\sigma_{1}^{2}+\sigma_{2}^{2}}}$$

and

(20) $$\sigma_{3}=\sqrt{\sigma_{1}^{2}+\sigma_{2}^{2}.\;}$$

A repeated standard deviation change from $\sigma_{1}$ to $\sigma_{3}$ with a stable $\sigma_{2}$ led to a quasilinear RMS roughness increase after sufficient iterations; hence, a nonconvergence RMS roughness value for the entire surface. To obtain convergent RMS roughness, a Markov chain was applied to compute a Gaussian-type random height distribution series with stationary initial and target surface background RMS. The background height variation series was calculated using the Metropolis–Hastings method [37] for a normal distribution to satisfy the convergent RMS roughness behavior within the scratch-free position during polishing. Silica abrasive particles were randomly selected from the ASD curve and placed on a silicon surface within a 500 × 500 pixels area. The particles moved at the same velocity in a random direction. After the mechanical process, the scratched position was Gaussian blurred with the background morphology offered by the Markov chain to simulate the chemical material removal process.

## 3. Experimental Setup

**Figure 9:**
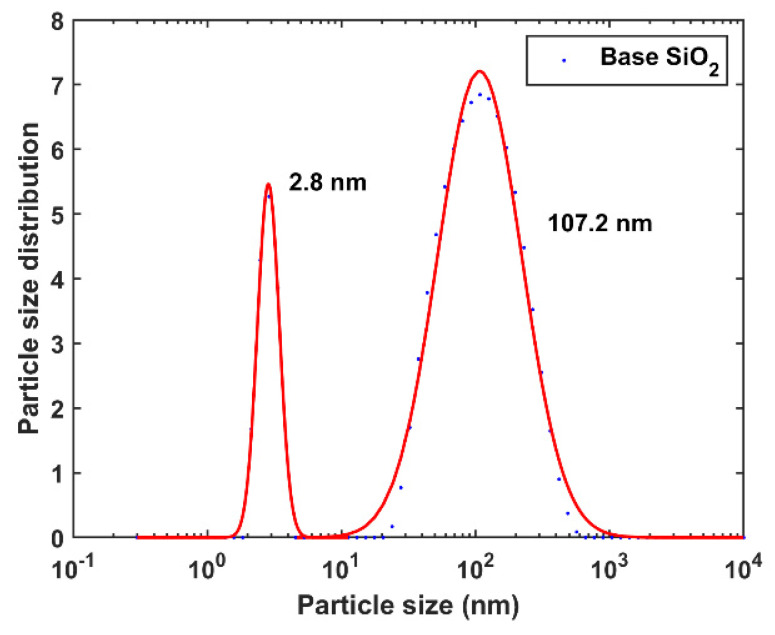
SiO_2_ ASD test by DLS, with a Gaussian-type fitting mode (red solid line); peaks at 2.8 and 107.2 nm for the bimodal ASD curve.

Colloidal SiO_2_ (JN-30, Qingdao Jiyida, Qingdao, China) was adopted as the polishing slurry. The silica colloid was diluted to 15 wt.% by ultrapure (UP) water (conductivity 1.12 μS/cm). The slurries were magnetically stirred to avoid slurry particle agglomeration during polishing, which could possibly influence the ASD. The ASDs for the polishing slurry were measured by using dynamic light scattering (DLS) techniques (Zetasizer, Malvern Panalytical, Malvern, UK; sizes ranging from 0.3 to 10^4^ nm). The results and fitting curves based on Equation (10) are shown in Figure 9. The ASD of the commercial colloidal silica was bimodal, with peaks at ~3 and 107 nm.

**Figure 10:**
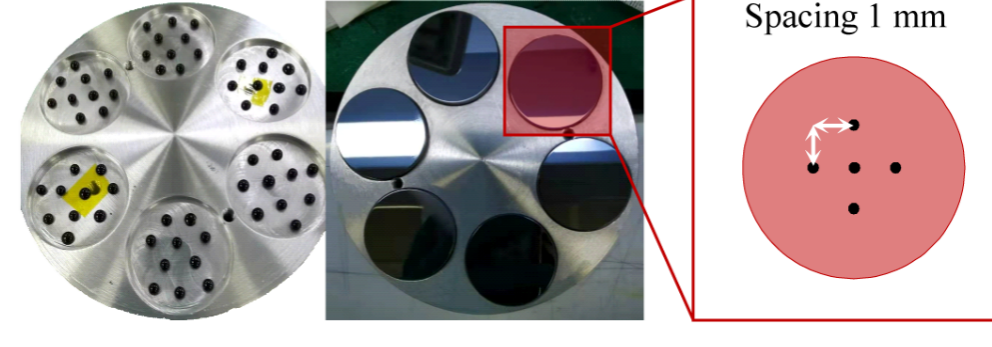
Pitch-button blocked workpieces.

Monocrystalline Si<111> wafers (diameter: 30 mm; thickness: 5 mm) were used as the workpiece. Six pieces of silicon wafers were pitch-buttons blocked on an aluminum connector and coarsely ground using diamond abrasive particles (Microgrit 10, 14, and 28 T) on a pig iron lap to obtain a quasiflat coplanar surface (Figure 10). For pad preparation, a polyurethane pad (LP66, Universal Photonics, New York, NY, USA) was attached to an aluminum polishing lap (diameter: 300 mm; thickness: 20 mm) by using hot melt glue and placed upside down on a flat marble slab to obtain a fully fit no-bubble surface. A 10 mm square pattern with 2 mm wide V-grooves having a depth of 3 mm was engraved on the pad for slurry flow. The workpieces were polished by using pure silica colloid slurry on a polyurethane pad with an applied load of 8 N and a slurry flow rate of 10 mL/min. After sufficient polishing time, the workpieces were rinsed with UP water. The polishing parameters used are listed in Table 1.

**Figure 11:**
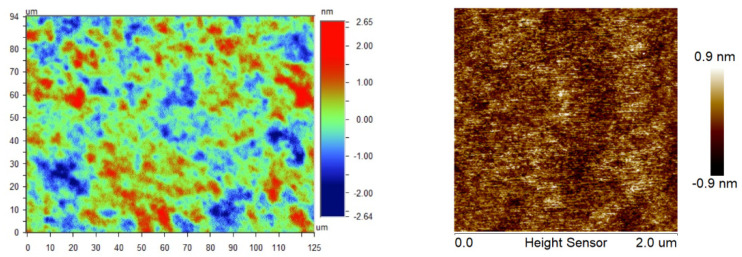
Si<111> surface morphology after CMP by performing profilometry within 125 μm × 94 μm (**left**) and AFM within 2 μm × 2 μm (**right**).

The surface morphology was tested using profilometry (50×, scan area of 640 × 480 pixels within 125 μm × 94 μm, narrow-band green light, ContourGT-X3, Bruker™, Billerica, MA, USA) and atomic force microscopy (AFM) (tapping mode, scan area of 256 × 256 pixels within 2 μm × 2 μm, AFM Dimension Icon, Bruker™, USA). The typical surface morphology results are shown in Figure 11, and the RMS roughness over five repetitions of the polishing experiments is listed in Table 2. The size of pixels for profilometry and AFM in the x–y direction is 20 μm and 7.8 nm, respectively. A typical scratch width in CMP is several nanometers, based on Section 2.1. It is much smaller than the pixel size of profilometry, so compared with AFM, the surface topography measured by profilometry is scratch-free. Additionally, both results show orange-peel structures regardless of the field of view, indicating that orange peel has fractal properties. The fractal surface analysis is further analyzed in Section 4.1.

## 4. Results and Discussion

### 4.1. Verification of the Micropolishing Model and Surface Morphology Evolution

**Figure 12:**
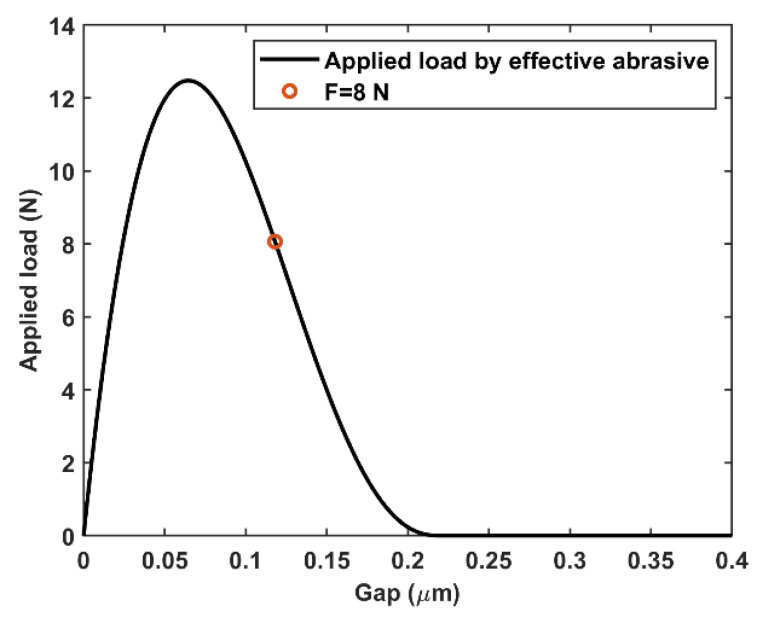
Applied load as a function of gap calculated by using Equation (12). As described in Section 3, 8 N was adopted as total applied load, and the gap value was 0.1185 μm (marked by an orange circle), based on the curve.

Although the experimental ASD curve is bimodal with two peak centers at 2.8 and 107.2 nm, most of the abrasives are located at the second peak. For simplicity, the first peak was ignored in the simulation. With an $r_{0}$ of 107.2 nm for the silica slurry adopted in the experimental setup (Figure 9) and pad, abrasive, and workpiece parameters summarized in Table 3, the calculated applied load as a function of h by using Equation (12) is shown in Figure 12. Before the load by the effective abrasive reached ~12 N, the gap decreased with an increase in the applied load. The maximum applied load was obtained at ~0.06 μm. A load larger than this limit causes direct contact with the workpiece pad; hence, the gap and nondirect contact surface areas covered by abrasives decreased. Because the slurry volume entering the gap is linear to the product of the gap and nondirect contact surface area, the number of abrasive particles in the gap decreased. Therefore, for loads larger than the limit, the load applied by the abrasive decreased with a decrease in the effective abrasive quantity.

The gap was 0.1185 μm for an applied load of 8 N. Gap h is two orders of magnitude larger than the first ASD peak center, indicating that the abrasives located at the first ASD peak did not contribute to the mechanical MRR. The mechanical material removal caused by the microscratching process can be obtained by calculating the width and depth distribution of the scratches. The scratched position was then Gaussian blurred, as explained in Section 2.2, with the normalization method presented in Section 2.3, to simulate the total material removal after one pass. The surface morphology evolution and final surface morphology after a sufficient polishing time can be achieved by continuous iterations of the procedure.

To test the validity of the micropolishing model on the RMS roughness behavior, it was necessary to compare the simulated surface roughness with the experimental results under the same polishing conditions. The mechanical parameters adopted in the simulation for Si<111> are listed in Table 3.

**Figure 13:**
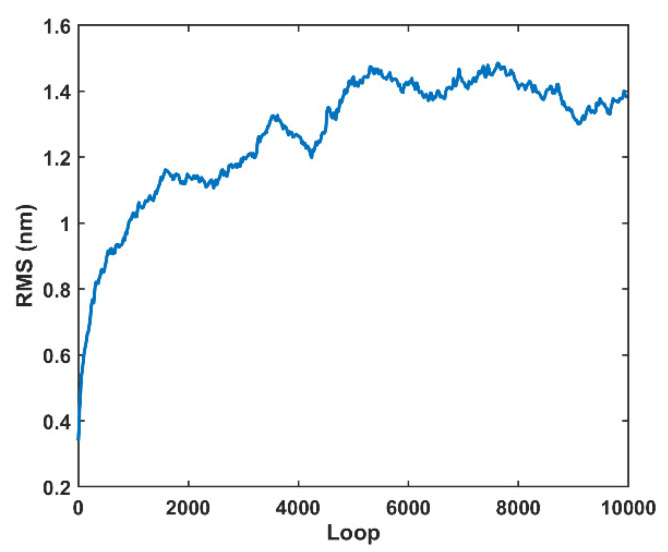
Simulated surface roughness for monocrystalline silicon after repolishing with pure colloidal silica slurry for 10,000 iterations.

The simulated surface RMS roughness evolution was analyzed within 10,000 iterations (Figure 13). An RMS roughness convergence of ~1.4 nm was observed after 5000 loops with a random fluctuation, which was the RMS roughness convergence point for the simulation. The RMS roughness over five repetitions of the polishing experiments was measured as 0.6–0.9 nm by employing profilometry and 0.2–0.4 nm by performing AFM, which has the same dimension as the simulated surface. During the calculation of penetration depth of slurry abrasive, the displacement difference at load/unload due to permanent plastic deformation was ignored, which may explain why the simulated RMS is slightly larger than those obtained by profilometry.

**Figure 14:**
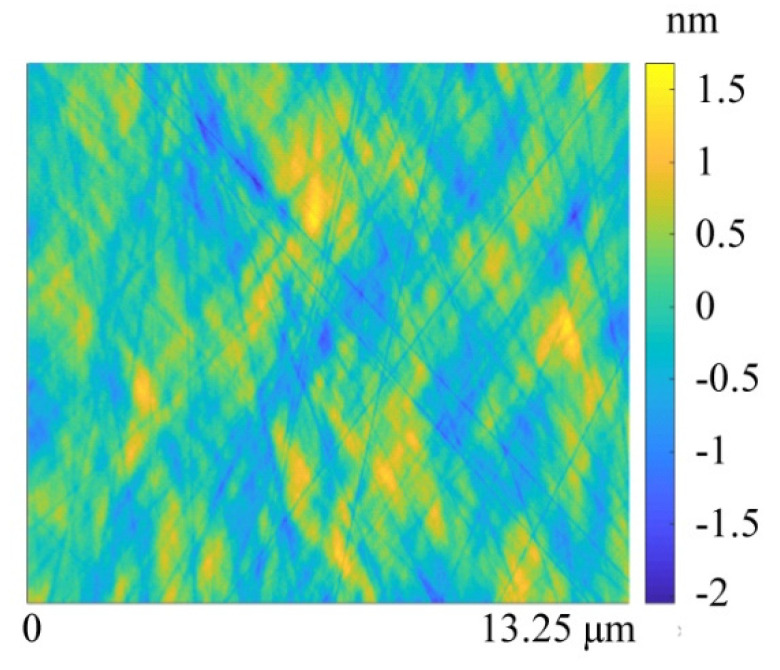
Simulated surface morphology for monocrystalline silicon with pure colloidal silica slurry within a 13.25 μm × 13.25 μm range for sufficient polishing iterations; the sampling points are 500 × 500 in the x–y direction.

The surface morphology after the RMS roughness convergence point is shown in Figure 14. The simulated surface morphology has features similar to the experimental results obtained by profilometry and AFM, as shown in Figure 11. Scratches and textures similar to orange peel, owing to mechanical scratching and exposure of grain boundaries formed during annealing, can be clearly observed in both experiments.

The simulation and experiment targeted different spatial periods. A direct comparison of the surface height information was not appropriate for morphology evaluation. This was proved by the RMS roughness deviation with profilometry and AFM for the same silicon workpiece (Figure 11). Additionally, surface uniformity is another fundamental consideration in evaluating the correlation between simulation and experiment. The PSD theory [38] was thereby adopted for morphology uniformity in different spatial periods. The 1D PSD of an isotropic fractal surface obeys the inverse power law by using fractal surface theory:

(21) $$S_{1}\left(f_{x}\right)=\frac{K_{n}}{f_{x}^{n}}=\frac{S_{1}\left(1\right)}{f_{x}^{n}},$$

where K denotes the spectral density, $S_{1}$ denotes the PSD value for each spatial frequency $f_{x}$, and $S_{1}\left(1\right)$ is the PSD value for $f_{x}=1$. The log–log plot for the PSD is linear with a slope value −n.

**Figure 15:**
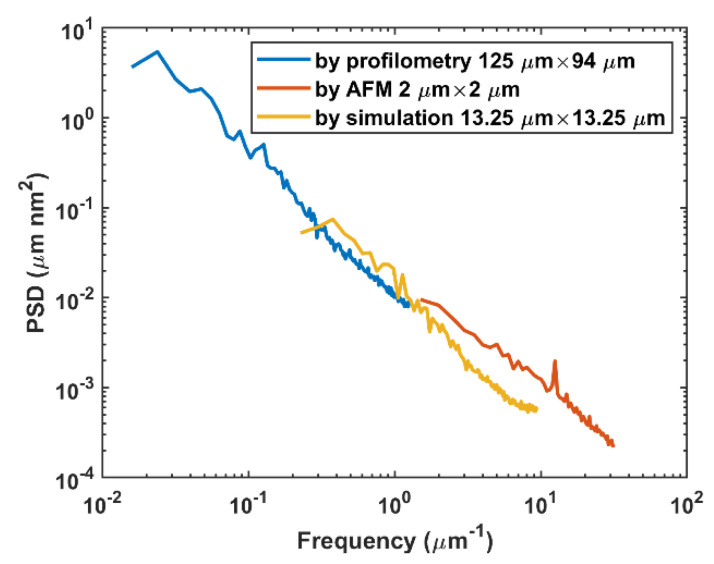
PSD for profilometry (125 μm × 94 μm, 640 × 480 sampling points), AFM (2 μm × 2 μm, 256 × 256 sampling points), and simulated surface (13.25 μm × 13.25 μm, 500 × 500 sampling points) for Si<111>.

One-dimensional-surface PSD curves from the profilometer, AFM, and simulation were calculated using PSD theory [38] and plotted in Figure 15. The adopted simulating area was 13.25 μm × 13.25 μm within 500 × 500 sampling points, and the PSD curve covered spatial periods for the profilometer and AFM. The three spatial periods correlated well with the omission of frequency at both ends of the three curves, caused by the field of view, lateral resolution, and white noise [3,39,40]. Additionally, three PSD curves follow the same linear relation with double-logarithmic coordinates, which indicates the three surfaces are isotropic with the same fractal dimension. Linear fitting for the PSD curve of AFM follows:

(22) $$\log\left(S_{1}\right)=-1.28\log\left(f_{x}\right)-3.87,$$

with R-square equaling 0.975. For profilometry and simulation, similar linear fitting functions were observed. According to the research by Chen et al. [41], we calculated the fractal dimension, which is ~2.6. The PSD curve of the simulated surface provided an adjustable spatial frequency between 10^–1^ μm^–2^ and 10^2^ μm^–1^ by changing the ratio of the contact circle radius $a_{i}$ over the pixels or by increasing the number of pixels. Because of the consistency in the PSD curve, the PSD for other spatial frequency regions can be extrapolated according to the fitting result. Therefore, the pixel resolution can be enlarged for surface morphology predictions on different observation scales at any polishing time.

### 4.2. Engineering Application of the Model

The ultrasmooth monocrystalline silicon mirror fabrication is a promising application for CMP. For investigations on CMP, high polishing efficiency and surface quality are often the most concerning issues. Although traditional experiment-based process improvements can achieve good polishing accuracy, a lack of knowledge of the material removal mechanism leads to long-term attempts and is often costly. In addition, the uncertainty of end-process surface morphology limits the application of CMP toward deterministic fabrication. In this paper, the effect of applied load, elastic–plastic behavior of materials, ASD, abrasive concentration, and chemical reaction rate determined by slurry components is evaluated theoretically. The micropolishing model established combines chemical erosion with mechanical scratching to predict morphology evolution on the microscale silicon surface. The simulated surface morphology is adaptable to various surface quality assessment standards because it contains the same information as the actual silicon surface. The model also provides openness with different workpiece and pad materials, provided their elasticity and plasticity match the assumption of the model.

In addition to end-process surface morphology predictions, the model can also be used to balance the polishing accuracy, efficiency, and processing cost. By analyzing Figure 5 and Figure 6, a higher slurry concentration or chemical reaction rate leads to a higher polishing efficiency before abrasive saturation. Increasing the applied load is another approach to achieve high efficiency, but the roughness worsens as scratches are more profound on the workpiece. An even higher applied load brings direct contact with the workpiece pad, which may cause severe damage to silicon mirror and polishing equipment. With the simulated applied load under different gaps, which in our case is the curve shown in Figure 12, maximum load on workpiece can be fixed to avoid this damage.

## 5. Conclusions

In this study, chemical and mechanical processes were studied by using elastic–plastic deformation and wet chemical etching based on microscopic material removal characteristics during CMP for a silicon workpiece. A micropolishing model was developed to predict microscale surface morphology during CMP. The predicted silicon mirror surface morphology captured salient features, such as microscratches and textures similar to orange peel, with the PSD curve and RMS roughness results being consistent with the experimental results measured by profilometry and AFM. The results imply valuable insights into the mechanism and prediction of microscale surface morphology evolution. These insights include: (1) The gap between the workpiece and pad was found to depend on the abrasive size distribution and applied load, which in turn determined the size of the abrasive that can generate effective mechanical removal. (2) The chemical reaction during CMP, which was previously considered to assist mechanical removal or to have a similar effect to mechanical material removal, was quantified based on scratch widening from the Gaussian-fitting wet chemical etch model and time-dependent background height transition. (3) Chemical and mechanical material removal processes were linked through the abrasive particle number and chemical etching rate to establish a micropolishing model with convergent RMS roughness evolution. (4) The PSD curves calculated from simulation and experiments indicate that the surface is fractal in mid- to short spatial frequency, which means the material removal mechanism in this region is consistent during CMP. The increased understanding of the CMP mechanism obtained from this model can be used for the impact quantification of different polishing factors and further optimization of the polishing process.

## Figures and Tables

**Table 1:** Polishing parameters of the polishing experiments.

Load (Pa)	Polishing Time (h)	Workpiece Rotation Velocity (rpm)	Pad Rotation Velocity (rpm)
1898	3	41	42

**Table 2:** The RMS roughness over a 5 point test of profilometry and 2 point test of AFM for 5 repetitions.

	RMS by Profilometry (nm)					RMS by AFM (nm)	
1	0.885	0.813	0.784	0.746	0.744	0.251	0.278
2	0.683	0.743	0.860	0.804	0.697	0.234	0.221
3	0.828	0.694	0.757	0.667	0.697	0.298	0.282
4	0.721	0.836	0.686	0.683	0.753	0.280	0.231
5	0.826	0.866	0.712	0.868	0.730	0.313	0.366

**Table 3:** Material and abrasive parameters for the micropolishing model.

*ν_m_*	0.26	*E_m_*	18.5 × 10^10^ Pa	Gap	0.1185 μm
*ν_p_*	0.17	*E_p_*	7.20 × 10^10^ Pa	ASD peak 1	2.8 nm
*ν_l_*	0.22	*E_l_*	3.14 × 10^8^ Pa	ASD peak 2	107.2 nm
Slurry concentration		15 wt.%		Applied load	8 N

## Data Availability

Not applicable.
